# Cardiometabolic Risk Increased in Working-Aged Adults During the COVID-19 Pandemic

**DOI:** 10.1089/met.2023.0044

**Published:** 2023-10-17

**Authors:** Maren S. Fragala, Fumika Matsushita, Zhen Chen, Lance A. Bare

**Affiliations:** Quest Diagnostics, Secaucus, New Jersey, USA.

**Keywords:** metabolic syndrome, employee health, blood pressure, prediabetes, health risk factors, workplace health

## Abstract

**Background:**

Public health measures necessary to mitigate the spread of coronavirus disease 2019 (COVID-19) impacted lifestyles and health practices. This multiyear cohort analysis of U.S. working-aged adults aims to evaluate the impact of the COVID-19 pandemic on metabolic syndrome and explores contributing factors.

**Methods:**

This longitudinal study (*n* = 19,543) evaluated year-to-year changes in metabolic syndrome and cardiometabolic risk factors through employer-sponsored annual health assessment before and during the COVID-19 pandemic using logistic mixed-effects model.

**Results:**

From prepandemic to pandemic (2019 to 2020), prevalence of metabolic syndrome increased by 3.5% for men and 3.0% for women, across all ethnic groups. This change was mainly driven by increased fasting glucose (7.3%) and blood pressure (5.2%). The increased risk of metabolic syndrome was more likely to occur in individuals with an elevated body mass index (BMI) combined with insufficient sleep or physical activity.

**Conclusions:**

Cardiometabolic risk increased during the COVID-19 pandemic compared with before the pandemic in a working-aged adult population, more so for those with a high BMI, unhealthy sleep, and low physical activity practices. Given this observation, identification of risk and intervention (including lifestyle and medical) is increasingly necessary to reduce the cardiovascular and metabolic risk, and improve working-aged population health.

## Introduction

Public health efforts necessary to mitigate the transmission of the coronavirus disease 2019 (COVID-19) had unintended and adverse consequences on health. While stay-at-home orders, site closures, and social distancing were necessary to mitigate the spread of COVID-19, emerging evidence^1–4^ suggests significant health consequences of the pandemic, beyond the virus itself. Fear of contracting the virus combined with government mandates created social isolation and impacted lifestyles, behaviors, and mental health.^4^ The impact of social isolation during the pandemic may be substantial, particularly regarding psychological consequences^5^ and cardiometabolic health.^1,6,7^

Cardiometabolic risk is prevalent in the U.S. population. Approximately 1/3 of U.S. adults have metabolic syndrome,^8,9^ a cluster of three or more of five cardiometabolic risk factors/metabolic dysregulations including insulin resistance, atherogenic dyslipidemia (high-density lipoprotein [HDL] cholesterol and triglycerides), central obesity, and hypertension.^10^

Metabolic syndrome is associated with increased risk of cardiovascular disease, diabetes,^11^ and all-cause mortality.^11,12^ It is costly to the health care system,^13^ as patients with metabolic syndrome cost ∼60% more per person per year, and ∼24% more per risk factor than those without metabolic syndrome.^13^ Importantly, metabolic syndrome is modifiable where cardiometabolic risk can be reduced with intervention. As cardiovascular disease is the leading cause of morbidity and mortality worldwide, understanding the population prevalence and associated risks is essential to reducing the disease burden.

The accumulation of stressful life events has been associated with obesity, insulin resistance, elevated triglycerides, and increased odds of having metabolic syndrome.^14^ Yet, the immediate impact of the COVID-19 pandemic on population cardiometabolic health is largely unknown. A recent systematic review suggests that COVID-19 has exacerbated risk factors for obesity and is likely to worsen obesity rates.^15^

Moreover, some data indicate disruptions in eating behaviors,^4^ physical activity,^4^ sleep,^4^ body weight, and blood pressure as a result of the pandemic period.^1,4,16–18^ The pandemic's impact on health has shown some evidence of disproportionately impacting some segments of the population, including women^1^ and individuals with obesity.^4^ Yet, what remains to be seen is whether this pandemic is exacerbating the growing metabolic disease burden. To date, no studies have assessed the direct impact of the COVID-19 pandemic on metabolic syndrome in a multiyear cohort of U.S. working-aged adults. Therefore, the objectives of this article are to fill in this gap by describing the effects of the COVID-19 pandemic on metabolic syndrome and cardiometabolic risk, and to explore contributing factors in vulnerable segments of the population.

## Methods

This retrospective longitudinal study included a cohort of 19,543 employees (81.3%) who remained working largely onsite to provide essential services during the pandemic period and their spouses/partners (18.7%)—working-aged adults—who completed an employer-sponsored annual health assessment operated by Quest Diagnostics, with year-end evaluations (September–November) in 2018, 2019, 2020, and 2021, which included biometric measurements, venipuncture blood draw, and a self-reported health risk questionnaire.

Preliminary analyses revealed no differences between employee and spouse/domestic partner participants in demographics (age, sex) and body mass index (BMI) distributions. Thus, both were included in the analysis to increase sample size. Three study periods were defined: prepandemic period (2018–2019), transition period from prepandemic to the first year of pandemic (2019–2020), and pandemic period (2020–2021); transition period was the focus for this study, with prepandemic and pandemic periods being two control periods to compare with the transition period. All participants were at least 18 years old in 2018, and all women who were pregnant during the study period were excluded from the study analysis.

A metabolic syndrome “score” was calculated for all participants in each year of the study. The score, on a scale from 0 to 5, indicated the number of abnormal/higher risk cardiometabolic risk factors: high fasting glucose (≥100 mg/dL), hypertension (systolic blood pressure [SBP] ≥130 mm/Hg or diastolic blood pressure [DBP] ≥85 mm/Hg), hypertriglyceridemia (≥150 mg/dL), large waist circumference (>35 inches for women; > 40 inches for men), and low HDL cholesterol (<50 mg/dL for women; <40 mg/dL for men).^19^ Being consistent with the current literature and industry standard, a participant with a metabolic syndrome “score” ≥3 was considered having metabolic syndrome in this study.

Sleep duration in hours per day and physical activity level per week were self-reported in a health risk assessment each year.

The probability of having metabolic syndrome (≥3 abnormal/higher risk cardiometabolic risk factors) versus ≤2 abnormal/higher risk cardiometabolic risk factors was compared between the 2 years in each of three study periods: prepandemic, transition period, and pandemic period. Probabilities were estimated in nested models by including additional covariates in a larger/outer model: adjusted for age and sex only (model 1), adding BMI to model 1 (model 2), adding race to model 2 (model 3), and adding sleep duration and weekly exercise level, one at a time, to model 3 (model 4).

For the main objectives of this study, probabilities from models 2 to 4 were only compared between 2019 and 2020 for the transition period. In addition to looking at 2-year clusters in the three periods as defined in the study design, a sensitivity analysis on model 1 (age and sex adjusted) with time variable including all 4 years was conducted to examine if a similar effect on the probability of metabolic syndrome was observed in the transition period (2019–2020).

The probability of having high-risk results in each of five cardiometabolic risk factors was estimated to determine the “dominant” factors that drove the observed differences in the probability of having metabolic syndrome, without and with adjustment for age and sex.

Logistic mixed-effects models for repeated measures were fitted to assess the adjusted effects of time (pandemic vs. prepandemic), age, sex, BMI, race, sleep duration, and exercise level on the outcomes, metabolic syndrome (score ≥3 versus score ≤2), and each of cardiometabolic risk factors (abnormal/higher risk results vs. normal/lower risk results). The SAS procedure NLMIXED was used to estimate the parameters and calculate the probability of the outcomes. *P* values <0.05 were considered statistically significant.

Statistical analyses were performed using SAS Studio 3.6 on SAS version 9.4. (Cary, NC). This study was deemed exempt by the WCG Institutional Review Board based on federal regulation 45 CFR Parts 46 and 164. The WCG Institutional Review Board is an independent ethical review board formed by the integration of the Western Institutional Review Board with Copernicus Group IRB, New England IRB, Aspire IRB, and Midlands IRB in 2020.

## Results

A total of 19,543 participants met study criteria and were included in the analysis. In this study population, mean age in 2018 was 47.3 (±standard deviation [SD] 10.9) years, and 12,152 (62.2%) were women.

During the transition period, prevalence of metabolic syndrome (score ≥3) increased by 3.1% (19.7% to 22.8%) (*P* < 0.001) from 2019 to 2020 (Table 1). However, in both the prepandemic period (2018–2019) and the pandemic period (2020–2021), prevalence of metabolic syndrome decreased by 0.7% (20.4% to 19.7%) and (22.8% to 22.1%), respectively (both *P* < 0.05) (Table 1).

**Table 1.** Characteristics of Participants Who Had Metabolic Syndrome (Score ≥3) or Were in the Higher Risk Group for Each Cardiometabolic Risk Factor, 2018–2021

	2018	2019	2020	2021
Metabolic syndrome	3,980 (20.4%)	3,842 (19.7%)^*^	4,456 (22.8%)^***^	4,315 (22.1%)^*^
Age, mean (SD)	49.7 (10.2)	50.8 (10.1)	51.6 (10.1)	52.8 (10.1)
Sex				
Women	2,228 (18.3%)	2,177 (17.9%)	2,536 (20.9%)^***^	2,450 (20.9%)^*^
Men	1,752 (23.7%)	1,665 (22.5%)^*^	1920 (26.0%)^***^	1865 (26.0%)
BMI, kg/m^2^				
BMI <30	1,167 (9.4%)	1,075 (8.7%)^*^	1,349 (11.1%)^***^	1,313 (10.8%)
BMI 30.0–34.9	1,229 (32.7%)	1,196 (31.2%)	1,371 (35.0%)^***^	1,353 (33.8%)
BMI ≥35.0	1,576 (48.5%)	1,562 (47.5%)	1,722 (51.3%)^***^	1,641 (49.3%)
Race/ethnicity				
White	1,586 (21.6%)	1,567 (21.4%)	1,777 (24.2%)^***^	1,756 (23.9%)
Black	460 (17.1%)	442 (16.5%)	538 (20.0%)^***^	484 (18.0%)^*^
Hispanic	486 (22.6%)	436 (20.3%)^*^	509 (23.7%)^***^	485 (22.5%)
Asian	419 (14.9%)	411 (14.6%)	494 (17.6%)^***^	497 (17.7%)
Sleep hours per day				
<6	778 (25.1%)	1,301 (21.8%)	1,472 (26.1%)^***^	1,467 (24.7%)
6–7	2,347 (19.6%)	1,292 (19.1%)^*^	1,452 (22.0%)^***^	1,396 (20.1%)
>7	709 (18.2%)	1,110 (17.8%)	1,385 (20.5%)^***^	1,274 (20.0%)
Exercise level per week				
0 time	1,555 (28.3%)	1,461 (26.8%)	1,645 (31.1%)^***^	1,622 (30.2%)
1–2 times	1,197 (20.8%)	1,242 (20.7%)	1,421 (23.9%)^***^	1,366 (23.1%)^*^
3–4 times	340 (15.6%)	305 (15.1%)	396 (18.9%)^**^	332 (16.1%)
≥5 times	142 (11.6%)	124 (10.5%)	191 (14.3%)^*^	153 (11.7%)
Cardiometabolic risk factor: higher risk group threshold				
Blood pressure: SBP ≥130 mm/Hg or DBP ≥85 mm/Hg	6,245 (32.0%)	6,449 (33.0%)^**^	7,457 (38.2%)^***^	7,585 (38.8%)
Fasting glucose: ≥100 mg/dL	4,597 (27.5%)	4,462 (26.7%)^*^	5,661 (33.9%)^***^	5,223 (31.2%)^***^
HDL cholesterol: <50 mg/dL for women; <40 mg/dL for men	5,399 (27.6%)	4,895 (25.1%)^***^	4,893 (25.0%)	4,535 (23.2%)^***^
Waist circumference: >35 inches for women; >40 inches for men	6,668 (34.1%)	6,661 (34.1%)	6,825 (34.9%)^**^	6,974 (35.7%)^**^
Triglyceride: ≥150 mg/dL	4,335 (22.2%)	4,309 (22.1%)	4,544 (23.3%)^***^	4,614 (23.6%)

*P* value comparing between index year and previous year using McNemar's test, except for age: ^***^ <0.001 ^**^ <0.01 ^*^<0.05.

*N* (%) are reported unless indicated otherwise.

Total # of participants is 19,543, except for BMI (*n* = 19,478), race (*n* = 14,989), sleep hours per day (*n* = 18,987), exercise level per week (*n* = 14,654), and fasting glucose (*n* = 16,732).

BMI, body mass index; DBP, diastolic blood pressure; HDL, high-density lipoprotein; SD, standard deviation; SBP, systolic blood pressure.

During the transition period, prevalence of metabolic syndrome increased by 3.5% in men and 3.0% in women (*P* < 0.001). Similarly, prevalence of metabolic syndrome increased across all race/ethnicity groups: 3.6% among Black participants, 3.4% among Hispanics, 3.0% among Asians, and 2.9% among Whites (all *P* < 0.001) (Table 1). During the same period, prevalence of metabolic syndrome increased by 2.4% in participants with BMI <30 kg/m^2^, 3.8% in those with BMI between 30.0 and 34.9 kg/m^2^, and 3.9% in those with BMI ≥30 kg/m^2^ (all *P* < 0.001) (Table 1).

Suboptimal lifestyle practices including insufficient sleep and lower levels of physical activity were associated with a higher prevalence of metabolic syndrome. Over the transition period, prevalence of metabolic syndrome increased by 4.3% among participants who reported either <6 hours sleep and those who reported participating in no physical activity (both *P* < 0.001) (Table 1).

**FIG. 1.**
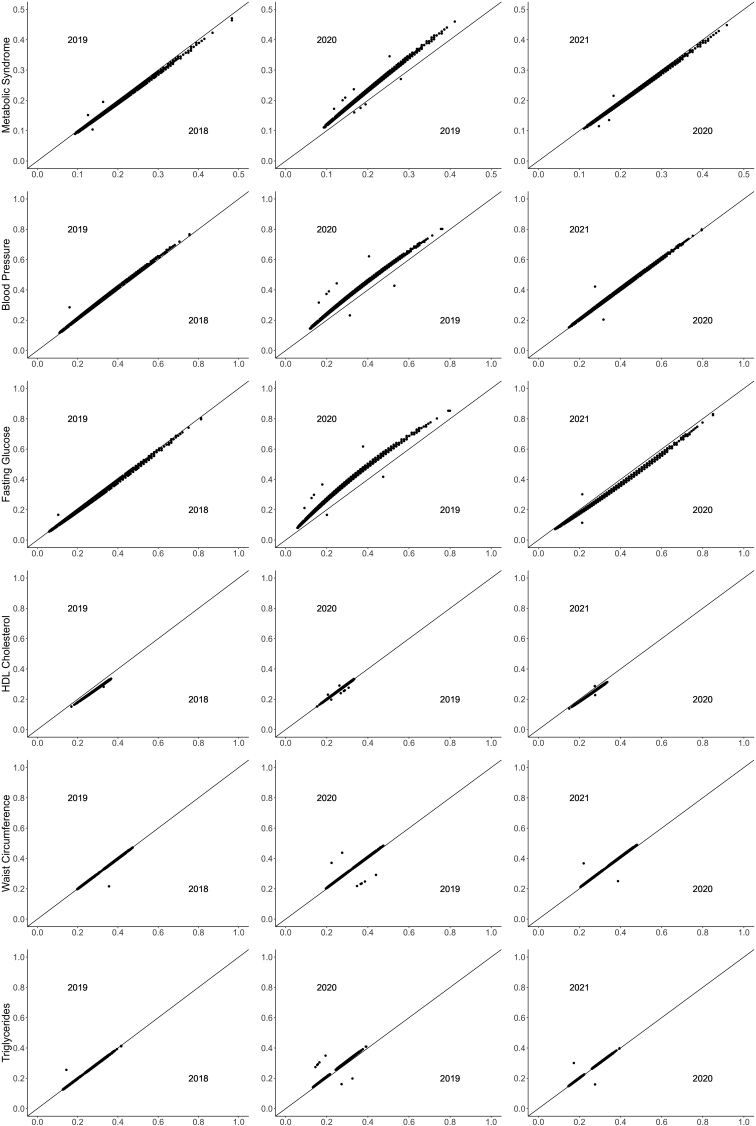
Probability of metabolic syndrome and higher risk cardiometabolic risk factors by 2-year cluster (2018–2019, 2019–2020, 2020–2021). All *P* values <0.001 in mixed logistic regression models, comparing index year (2019/2020/2021) and previous year (2018/2019/2020), modeling the probability of metabolic syndrome or being in the higher risk group for each cardiometabolic risk factor, adjusted for age and sex.

In Figure 1 based on model 1, during the transition period, probability of having metabolic syndrome increased from 2019 to 2020, after adjusting for age and sex (*P* < 0.001). Within the prepandemic period (2018–2019) and pandemic period (2020–2021), probability remained the same (Fig. 1). Examination of all 4 years of data together through a sensitivity analysis on model 1 (age and sex adjusted) yielded an equivalent effect on the probability of metabolic syndrome between 2019 and 2020 (*P* < 0.01).

Using the same multivariate model adjusted for age and sex, only two cardiometabolic risk factors, fasting glucose and blood pressure, showed a similar increase in year-to-year changes in the transition period from 2019 to 2020; the other three cardiometabolic risk factors including waist circumference, HDL cholesterol, and triglycerides did not show an evident change over the 4 study years including the transition period (2019–2020) (Fig. 1).

**FIG. 2.**
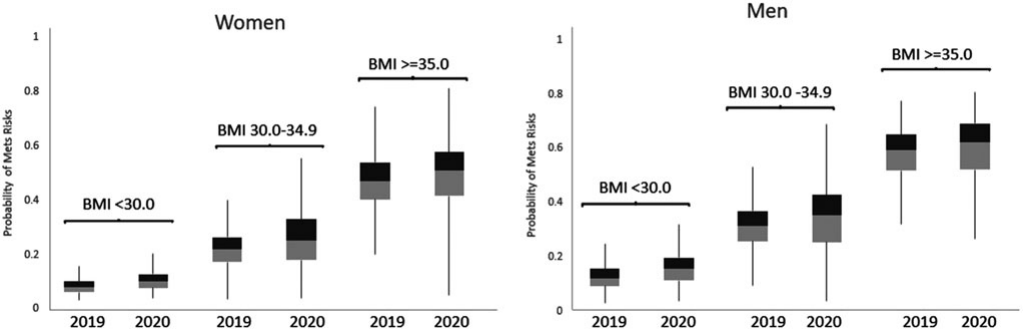
Probability of metabolic syndrome by sex and BMI 2019–2020. All *P* values <0.001 in mixed logistic regression modeling the probability of metabolic syndrome (2020 versus 2019), adjusted for age and sex. BMI, body mass index.

During the transition period, probability of having metabolic syndrome increased from 2019 to 2020, after adjusting for age, in both women and men and across all BMI categories (<30, 30–34.9, ≥35 kg/m^2^) (all *P* < 0.001) (Fig. 2). In this period, men had a higher probability of metabolic syndrome than women (*P* < 0.001); the median probability increased by 0.03–0.04 in men and by 0.02–0.03 in women, across three BMI categories. A higher BMI was associated with a higher probability of metabolic syndrome (*P* < 0.001); the median probability increased by 0.03 across all BMI categories from 2019 to 2020.

**FIG. 3.**
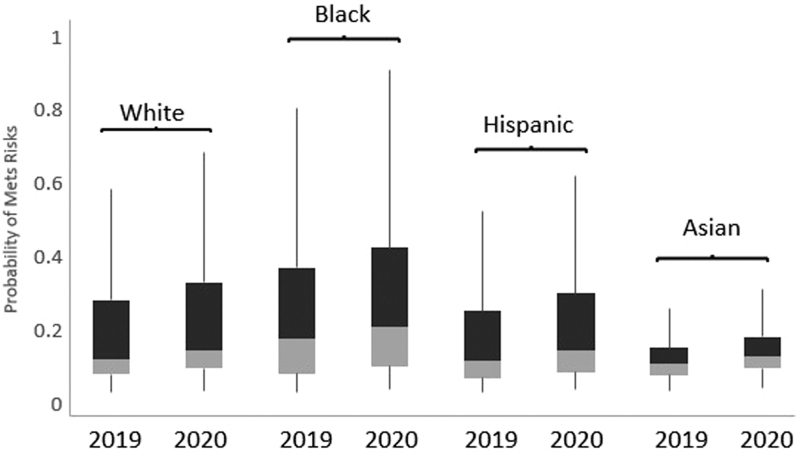
Probability of metabolic syndrome by race 2019–2020. All *P* values <0.001 in mixed logistic regression modeling the probability of metabolic syndrome (2020 versus 2019), adjusted for age, sex, and BMI.

During the transition period, probability of having metabolic syndrome increased from 2019 to 2020, after adjusting for age, sex, and BMI, in all ethnic groups (all *P* < 0.001) (Fig. 3). In this period, Black participants had the highest probability, followed by White and Hispanic, and then by Asian participants (*P* < 0.001); the median probability increased by 0.02 for Black, Hispanic, and White groups, and by 0.01 for Asians, from 2019 to 2020.

**FIG. 4.**
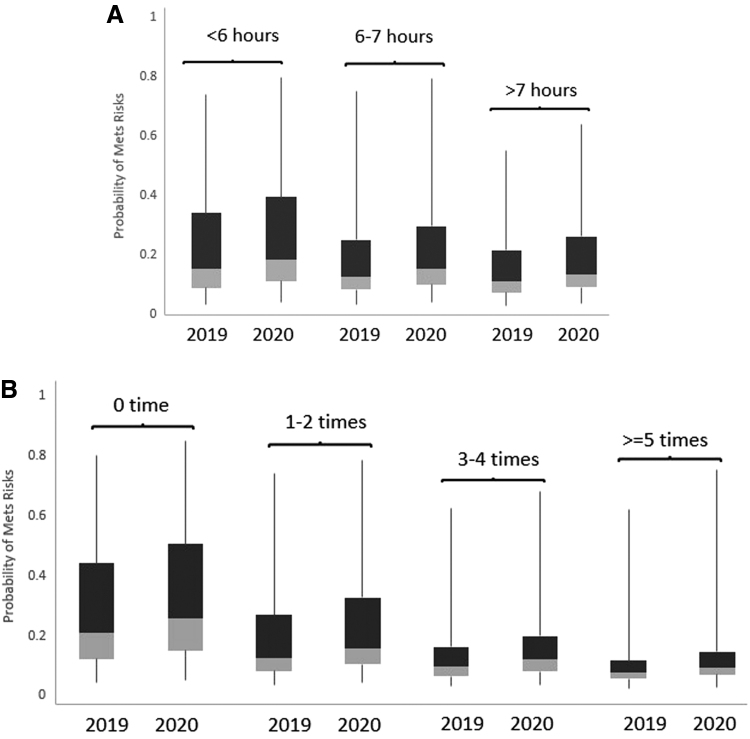
Probability of metabolic syndrome 2019–2020 by **(A)** sleep duration per night and by **(B)** weekly exercise level. All *P* values <0.001 in mixed logistic regression modeling the probability of metabolic syndrome (2020 versus 2019), adjusted for age, sex, BMI, and race.

During the transition period, probability of having metabolic syndrome increased from 2019 to 2020, adjusted for age, sex, BMI, and ethnicity, in all sleep duration categories (<6 hours, 6–7 hours, and >7 hours) (all *P* < 0.001) (Fig. 4A). In this period, the less sleep per night, the higher risk of metabolic syndrome (*P* < 0.01); the median probability increased by 0.02–0.03 across all three sleep duration categories from 2019 to 2020.

During the transition period, probability of having metabolic syndrome increased from 2019 to 2020, adjusted for age, sex, BMI, and ethnicity, across all exercise levels per week (0 time, 1–2 times, 3–4 times, and ≥5 times) (all *P* < 0.001) (Fig. 4B). A lower level of exercise was associated with a higher probability of metabolic syndrome (*P* < 0.001); the median probability increased by 0.05 for those participants who did not exercise at all, followed by 0.03 for one to two times, and then by 0.02 for three times or more, from 2019 to 2020.

## Discussion

We evaluated the impact of the COVID-19 pandemic on prevalence of metabolic syndrome in working-aged adults. The main finding of this analysis was that metabolic syndrome increased during the COVID-19 pandemic in this large U.S. population of working-aged adults. Although cardiometabolic risk has been increasing gradually in the U.S. population over the few decades,^8,20^ this study indicates measurable increases in risk during the COVID-19 lockdown period.

Previous research has similarly reported increases in cardiometabolic risk during the lockdown period in a smaller patient population^21^ and a Spanish population.^22^ To our knowledge, this is the first report to show increases in a large U.S. working-aged population after the onset of the COVID-19 pandemic.

Prevalence of metabolic syndrome observed in the population of employees and their spouses/partners was similar to the rates observed in the U.S. adult population, where the prevalence of metabolic syndrome is 34.7% (with similar rates observed in men [35.1%] and women [34.3%]).^8^ National population health trends have shown increases in metabolic syndrome prevalence over the last decade, especially among those aged 20–39 years (from 16.2% to 21.3%), women (from 31.7% to 36.6%), Asian participants (from 19.9% to 26.2%), and Hispanic participants (from 32.9% to 40.4%).^8^ The high and growing rates of metabolic syndrome in U.S. working-aged populations present a major public health burden as metabolic syndrome is associated with increased risk of cardiovascular disease and all-cause mortality,^11,12^ higher medical costs,^23,24^ and lower job performance.^25^

Increases in metabolic syndrome were more commonly seen in individuals with a BMI in the overweight and obese range. The association of high BMI with metabolic syndrome has been clearly established, where metabolic syndrome impacts ∼60% of U.S. adults with obesity versus only 5% with normal body weight.^26,27^ Reasons for the greater increase in risk in those with a higher BMI are unknown but may be attributed to greater burden of the pandemic in individuals with an overweight or obese BMI. Specifically, the deleterious health behavior impacts of social isolation and stress may have disproportionately affected individuals with obesity, as previous reports showed that weight gain during the COVID-19 pandemic was more commonly reported in individuals with obesity.^4^ Factors contributing may include declines in physical activity,^4^ poor nutrition,^4^ and psychological stress.^4,28^ Not only has higher perceived stress been associated with a higher BMI and obesity,^29^ but increases in anxiety scores during the pandemic have been reported to be greater in people with obesity.^4^

As social isolation is associated with more intense stress during everyday events^30^ and higher cortisol reactivity in response to stress leads to higher calorie consumption and preferences for sweet foods,^28^ the impact of the COVID-19 pandemic on lifestyle behaviors, body weight, and cardiometabolic risk factors may have presented an additional major public health burden.

Increases in metabolic syndrome were noted most commonly in men and women who reported sleeping <6 hours per night. The lockdown period was associated with a higher prevalence of sleep problems, impacting ∼18% of the general public.^31,32^ Sleep problems were especially common among health care workers (31%) and COVID-19 patients (57%).^32^ Moreover, the pandemic impacted sleep quality, where 43.8% reported worsened sleep quality.^4^ Previous research has linked insufficient sleep to cardiovascular and metabolic risk.^33,34^

Insufficient sleep has been associated with several health risks, including obesity, diabetes, hypertension, heart disease and stroke, and even premature death.^33,34^ Weight gain associated with insufficient sleep predisposes individuals to abdominal visceral obesity, and associated cardiovascular and metabolic risk.^35^ Thus, insufficient sleep appears to be a contributing factor to increases in metabolic syndrome likely through the mechanisms noted above.

Sleep deprivation has been reported to have a significant impact on metabolic and cardiovascular function,^36^ and this underappreciated risk factor deserves a full examination in large-scale population studies. Health interventions to mediate the cardiovascular and metabolic risk should evaluate and treat sleep disturbances, as appropriate.

Increases in metabolic syndrome were most commonly observed in men and women who reported participating in no physical activity. Physical activity modulates excess body weight,^37^ and is known to prevent and mitigate the impact of the metabolic syndrome.^38^ During the lockdown period, physical activity slightly decreased except in those with physical activity more than five times a week (increased by 1.7%, *P* < 0.001). Others similarly reported that sedentary leisure behaviors increased, while time spent in physical activity declined during the pandemic lockdown period.^4^ Insufficient physical activity appears to be a contributing factor to increases in the risk of metabolic syndrome. Thus, increasing physical activity is an important intervention to mitigate observed cardiometabolic risk increases, which is cost effective and underutilized.^38^ Moreover, the beneficial impact of physical activity on cardiovascular disease risk appears to even outweigh the negative impact of BMI in adults.^37^

Reasons for observed changes in health risks and behaviors may be attributable to social isolation and stress—both have unfavorable impacts on health and health behaviors.^29,39–41^ Social isolation adversely impacts health behaviors^39–41^ (including physical inactivity,^40,41^ poor diet,^40,41^ poor sleep quality,^39^ smoking,^41^ and use of psychotropic medications)^40^ and outcomes^39–41^ (including poor self-rated health,^40^ depression,^39,40^ unfavorable cardiovascular function,^39^ impaired immunity,^39^ impaired executive function,^39^ accelerated cognitive decline,^39^ altered hypothalamic pituitary-adrenocortical activity,^39^ a proinflammatory gene expression profile,^39^ multiple health problems,^40^ and earlier mortality).^39^

Future investigations are necessary to evaluate the relationship between stress factors and health risks. In addition, while the present investigation used fasting glucose as a measure of insulin resistance according to the existing criteria of metabolic syndrome,^10^ future evaluations should consider assessment of metabolic risk using hemoglobin A1C. Assessment of metabolic risk using hemoglobin A1C offers some advantages to fasting glucose, including nonfasting specimen collection and improved prediction of incident diabetes.^42,43^

Given the observation of increases in cardiometabolic risk in working-aged participants, screening and intervention (lifestyle and pharmaceutical) are increasingly necessary to reduce the cardiovascular and metabolic risk. Employees with metabolic syndrome have double the costs of those without any risk factors.^23,24^ Of the excess medical cost for individuals with metabolic syndrome, ∼20% is because of additional cardiovascular events and ∼80% because of the expense of higher prevalence of comorbidities, particularly cardiovascular disease and diabetes.^24^ Thus, prevention strategies including lifestyle interventions and drug therapy, if required, are needed to reduce the risk of cardiovascular disease^19^ and diabetes.^11^

Strategies to reduce the burden of metabolic syndrome may include lifestyle interventions such as Mediterranean diet^44^ and Diabetes Prevention Program intensive lifestyle interventions.^45^ Pharmaceutical interventions to manage the risk may include lipid lowering agents, antihypertensive agents, antidiabetic agents, heart failure drugs, and antiobesity therapies.^46,47^

## Conclusion

Metabolic syndrome prevalence increased in this working-aged adult population during the transition period from prepandemic to the first COVID-19 pandemic year of 2019–2020. Despite rising prevalence of metabolic syndrome in U.S. adults in recent years, the pandemic appeared to contribute to risk increases during year 1 due to disrupted lifestyles, lower physical activity, and sleep disturbances. Those with existing metabolic risk due to overweight and obesity appeared to be particularly vulnerable to increases in the risk of metabolic syndrome. Management of the growing risk of metabolic syndrome presents an increasingly urgent priority for employers to support the health of their workforce.

## Ethical Considerations

The analyses of this study were conducted according to the HIPAA Privacy Rule (Title 45 Code of Federal Regulations, Section 164.514e), which governs research conducted by covered health care entities and allows retrospective analysis using a limited data set without requiring approval of an institutional review board.

## Authors' Contributions

M.S.F. contributed to conceptualization (lead), writing—original draft (lead); F.M. performed data curation, analysis, data visualization, and writing; Z.C. assisted with conceptualization (supporting), development and design of methodology, reviewing and editing; L.A.B. supported with conceptualization (supporting), analysis supervision, reviewing and editing.
